# Cross-cultural adaptation and validation of the Amsterdam Instrumental Activities of Daily Living questionnaire short version German for Switzerland

**DOI:** 10.1186/s12955-020-01576-w

**Published:** 2020-10-02

**Authors:** Marina Bruderer-Hofstetter, Mark A. Dubbelman, André Meichtry, Florian Koehn, Thomas Münzer, Roos J. Jutten, Philip Scheltens, Sietske A. M. Sikkes, Karin Niedermann

**Affiliations:** 1 School of Health Professions, Institute of Physiotherapy, Zurich University of Applied Sciences, Katharina-Sulzer-Platz 9, 8401 Winterthur, Switzerland; 2 Department of Health Sciences and Medicine, University of Lucerne, Lucerne, Switzerland; 3 Alzheimer Center Amsterdam, Department of Neurology, Amsterdam Neuroscience, Vrije Universiteit Amsterdam, Amsterdam UMC, Amsterdam, The Netherlands; 4 Geriatrische Klinik St. Gallen, St. Gallen, Switzerland; 5 Department of Geriatrics and Aging Research, University Hospital and University of Zurich, Zurich, Switzerland

**Keywords:** Instrumental Activities of Daily Living (IADL), Assessment, Amsterdam IADL questionnaire, Cross-cultural validation, Elderly people, Mild cognitive impairment (MCI), Mild dementia

## Abstract

**Background:**

Instrumental Activities of Daily Living (IADL) limitations are associated with reduced health-related quality of life for people with mild cognitive impairment (MCI). For these people, the assessment of IADL is crucial to the diagnostic process, as well as for the evaluation of new interventions addressing MCI. The Amsterdam IADL Questionnaire Short Version (A-IADL-Q-SV) is an established assessment tool with good psychometric properties that has been shown to be robust to cultural differences in Western countries. The aims of this study were to: (1) cross-culturally adapt and validate the A-IADL-Q-SV for the German-speaking population of Switzerland; (2) investigate its cultural comparability; and (3) evaluate further psychometric properties.

**Methods:**

The A-IADL-Q-SV German was pretested on clinicians and participants in a memory clinic setting. The psychometric properties and cultural comparability of the questionnaire were investigated in memory clinic settings including participants with MCI or mild dementia, as well as participants with normal cognition recruited from the community. Item response theory (IRT) was applied to investigate measurement invariance by means of differential item functioning to assess item bias. Additionally, the test–retest reliability on scale level, the construct validity through hypothesis testing and the discriminant validity of the A-IADL-Q-SV German were evaluated.

**Results:**

Ninety-six informants of participants with normal cognition, MCI or mild dementia completed the A-IADL-Q-SV German. The basic assumptions for IRT scoring were met. No meaningful differential item functioning for culture was detected between the Swiss and Dutch reference samples. High test–retest reliability on scale level (ICC 0.93; 95% CI 0.9–0.96) was found. More than 75% of the observed correlations between the A-IADL-Q-SV German and clinical measures of cognition and functional status were found to be in the direction and of the magnitude hypothesized. The A-IADL-Q-SV German was shown to be able to discriminate between participants with normal cognition and MCI, as well as MCI and mild dementia.

**Conclusions:**

The A-IADL-Q-SV German is a psychometrically robust measurement tool for a Swiss population with normal cognition, MCI and mild dementia. Thus, it provides a valuable tool to assess IADL functioning in clinical practices and research settings in Switzerland.

*Trial registration* This study was registered retrospectively in July 2019 on ClinicalTrials.gov (NCT04012398).

## Introduction

Instrumental activities of daily living (IADL) comprise the complex tasks needed to live independently in society [1]. Within the context of cognitive decline, IADL were defined as, “Complex activities with little automated skills for which multiple cognitive processes are necessary” [2].

Mild cognitive impairment (MCI) is a transient health state between normal cognition (NC) and dementia [3]. People with MCI experience cognitive and physical functioning impairments [3] and IADL limitations are frequent [4]. The latter are associated with reduced health-related quality of life [5] and are one of the defining features distinguishing MCI from NC [6]. They are predictive of the future development of dementia, both for people with MCI and NC [7]. Therefore, IADL performance is an important aspect of early cognitive diagnostics [8].

Researchers are becoming increasingly aware of the importance of assessing IADL performance as a key outcome in intervention trials on older people with MCI and mild dementia (MD) [4]. Improvements in IADL performance make a treatment meaningful for patients [9]. Furthermore, besides quality of life and self-efficacy, IADL performance is a prioritised treatment outcome for people with MCI and their caregivers [10]. To adequately assess the efficacy and effectiveness of IADL interventions, and to allow for comparison between studies, assessment tools with good psychometric properties (e.g. reliability, validity, sensitivity to change) are needed. Ideally, they are also robust across different languages and cultures.

To date, no gold standard exists for the assessment of IADL performance. Different methods of measurement are applied, i.e. performance-based assessments, self-rated and/or informant-rated questionnaires [11]. For people with early cognitive decline, informant-based questionnaires are the most accurate and convenient form of assessment [12]. However, the face validity of older, although well-known, questionnaires has been questioned, since they do not include activities with respect to technical appliances (e.g. computer use) [11]. Additionally, commonly-used IADL questionnaires have poor psychometric properties [13] and lack in sensitivity when classifying healthy ageing, MCI and dementia [14]. Several self-reported and informant-reported IADL questionnaires have recently been developed to address these drawbacks. These questionnaires are sensitive to IADL limitations in the early stages of cognitive decline [4].

The informant-based Amsterdam IADL Questionnaire (A-IADL-Q) was developed to assess IADL functioning. It includes a wide range of IADLs covering all stages of cognitive decline in the setting of memory clinics [2]. The A-IADL-Q has been validated in a Dutch cohort and demonstrated good psychometric properties [15], as well as diagnostic value [16]. It was shown to be sensitive to capturing changes over time [17], and also to be robust across cultural differences in a comparison between different Western countries, with regard to culture, sex, age and education [18]. The European Joint Program for Neurodegenerative Diseases Working Group has recommended the use of the A-IADL-Q for research and clinical purposes [19]. The original A-IADL-Q contains 70 items, while the short version (A-IADL-Q-SV) contains 30 items [17]. The questionnaire has been translated into thirteen different languages, including German. The translation into German was made by ICON plc, a company specialising in the translation of clinical instruments (unpublished). The translation process followed the steps recommended by Beaton et al. [20]. This involved making two independent forward translations into the target language (i.e. German) followed by reconciliation into one version of the forward translation. Subsequently, two independent backward translations into the source language (i.e. Dutch) were made to check whether the intended meaning of the items, answer options and instructions had been retained. The translation process was finalized by a consensus meeting of the translators, the developer and translation project coordinator [20]. Although clinicians have already reviewed the translated German version, its cross-cultural validity in Switzerland has not yet been established.

Therefore, the aims of this study were to: (1) Adapt and validate the A-IADL-Q-SV German version cross-culturally, in order to be able to assess IADL performance in Switzerland of community-dwelling elderly people with NC, MCI and MD; (2) Further evaluate specific psychometric properties (i.e. measurement invariance, test–retest reliability, construct validity and interpretability).

## Methods

### Design

To obtain a final version of the A-IADL-Q-SV German version, we firstly pre-tested the translated questionnaire on clinicians and participants in a memory clinic setting to assess the comprehensibility of the translation, highlight any items that may be inappropriate at a conceptual level, and identify any other issues that may cause confusion, e.g. unclear wording [20, 21]. This final version of the A-IADL-SV German was then evaluated in an observational study with two measurement time points. Data from the first measurement time point were used to investigate measurement invariance, construct validity and discriminant validity. Data from both the first and second measurement time points were used to investigate test–retest reliability.

The study was approved by the responsible ethics committee (EKOS, BASEC-NR. 2017-02200) and was conducted in accordance to the Declaration of Helsinki.

### The A-IADL-Q-SV

The A-IADL-Q-SV contains 30 items and requires about 10 to 15 min for completion [22]. The questionnaire is adaptive and computerized, although it can also be administered on paper (with additional instructions necessary). In this study, the paper version of the questionnaire was used. All items are rated on a five-point scale, ranging from ‘no difficulty’ to ‘unable to perform’; scoring is based on item response theory (IRT) [15, 22]. The A-IADL-Q and A-IADL-Q-SV have been found to meet all the basic assumptions of IRT scoring, based on a Graded Response Model: (1) Unidimensionality, which implies that one underlying latent trait determines the items (in this case IADL functioning); (2) Local independence, meaning the independence of item responses, conditional on the latent trait; and (3) Monotonicity, meaning the probability of endorsing higher item categories as the trait level increases [15, 22]. The IRT latent trait levels were transformed into a ‘T-score’ that was calibrated to a memory clinic population, with a range from approximately 20 to 80, a mean of 50 and a standard deviation of 10, with higher ‘T-scores’ indicating better IADL functioning [15]. The A-IADL-Q-SV was translated into German; work on this translation was not published before. All 30 items of the German questionnaire are the same as in the original version, and are described in the Additional file 1: Table 1. The A-IADL-Q-SV German can be obtained from the developers after registration, and is free for use in all public health and non-profit agencies (https://www.alzheimercentrum.nl/professionals/amsterdam-iadl/).

### Participants and sample size

Community-dwelling older persons of age > 60 years and with NC, MCI or MD, together with their informants, were included in the study. Informants could be relatives, close friends or caregivers, who interacted closely enough with the participant to be able to respond to the questionnaire. Exclusion criteria for participants were: ‘Moderate to severe’ cognitive decline (based on the Mini Mental State examination (MMSE; < 20) for participants with MCI or MD, and the modified telephone interview for cognitive status (TICS-m; < 32) for participants with ND; Cognitive decline due to causes other than Alzheimer’s disease or vascular dementia, (e.g. neurological diseases, trauma, and people diagnosed with depression, alcohol or drug misuse). Participants with probable MCI or MD were recruited from two memory clinics in the German-speaking region of Switzerland (Geriatrische Klinik, St.Gallen; Psychiatrie St.Gallen Nord, Wil). General practitioners refer people with potential MCI or MD to a memory clinic for clarification of their cognitive complaints (i.e. dementia screening) as part of standard care. During these screening visits, a member of our study team gave people verbal and written information on the study, answered pending questions and obtained written informed consent. Participants with NC were recruited from the local community via flyers and advertisements distributed by the Pro Senectute St. Gallen organization and the Association of active older-persons in the city and region of St. Gallen. Interested persons were prompted to contact the study team by e-mail or telephone. A member of the study team then provided verbal information to these interested persons, answered pending questions and scheduled a phone call to check the eligibility criteria (e.g., TICS-m).

The targeted sample size to execute the cognitive debriefing/pretest was five clinicians and a minimum of five informants from people with MCI or MD to complete the A-IADL-Q-SV, with the option to recruit additional informants until no new issues or comments were raised [21]. The targeted sample size for the evaluation of the A-IADL-Q-SV German version was 100 participants, based on the proposed COSMIN recommendations [23]. Firstly, a sample size of 50 participants is recommended for test–retest analyses, including the calculation of intraclass correlation coefficients (ICCs) (two measurements, targeted ICC of 0.8 with width 0.2 of the 95% confidence interval) [23]. Secondly, a minimum of 50 participants is required (larger samples are recommended, e.g. 100 participants) for the investigation of the cross-cultural validity based on hypothesis testing by means of correlations [23].

### Procedures cognitive debriefing/pretest

Initially, five clinicians from a memory clinic were asked to give feedback on the A-IADL-Q-SV German. Issues discussed included answer options, activities or sentences, and the grade of difficulty. As a result, small adjustments were made and documented. Such adjustments included, e.g. the correction of spelling mistakes and grammatical inaccuracies, and specification of items (e.g. item 24: ‘operating devices’ into ‘operating electronic devices’).

Eight informants of people with MCI or MD completed the A-IADL-Q-SV German. The thinking-out-loud method was used, where informants were asked to write down their comments and issues on the relevance of each item, the applicability/meaning of the activities in Switzerland and the understandability of the questions. The results were reviewed to identify the necessity for translation modifications (e.g. rewording of items/response options). Additionally, the completed questionnaires were examined to detect high levels of missing items or single responses. Minor adjustments were again made to the questionnaire and fully discussed with the developer. Points of discussion included the specification of items, e.g. item 20 ‘work’ was supplemented with the specification ‘paid or unpaid’; or for item 11 ‘household appliances’ the possibility of complementing it with examples was discussed, but rejected because it may have influenced participants’ responses. Accordingly, a final version of the A-IADL-Q-SV German was obtained.

### Procedures validation and test–retest reliability of the A-IADL-Q-SV German

Measurements were performed in the memory clinics during the standard cognitive testing sessions for participants with MCI or MD. Each participant underwent an extensive cognitive screening procedure, including clinical and neuropsychological assessments, following international standards for dementia diagnosis [24]. During the same sessions, the informants completed the A-IADL-Q-SV German.

Interested participants from the community were contacted by telephone to check the inclusion and exclusion criteria. Thereafter, a cognitive impairment screening was performed, using the modified Telephone Interview for Cognitive Status (TICS-m) [25]. An education-adapted score of ≥ 32 points out of 50 points was required to qualify as not being subject to cognitive decline [26]. Their informants also completed the A-IADL-Q-SV German.

All informants were asked to complete the questionnaire a second time some 2 to 4 weeks later. Due to this short time interval, it was assumed that the cognitive status remains stable and that a deterioration in the IADL performance was very unlikely [27].

### Additional clinical assessments

The following additional clinical assessments were used in this study:

The *Mini-Mental State Examination *(*MMSE*)—assesses global cognition (score range 0–30), with higher scores indicating better cognitive performance [28]. The MMSE is the most widely used global cognitive screening tool in clinical and research settings with sound psychometric properties [19].

The *Clinical Dementia Rating* (*CDR*)—an assessment to stage the severity of dementia (score range 0–3), with higher scores indicating more severe stages of dementia [29]. The CDR is a recommended staging scale of dementia with high inter-rater reliability, good discriminant and concurrent validity [30].

The *Informant Questionnaire for Cognitive Decline in the Elderly* (*IQCODE*)*—*assesses cognitive decline based on questions regarding cognitive performance (score range 1–5), with higher scores indicating worse performance [31]. The IQCODE is widely used as a screening test for dementia. It has been shown to measure a single factor of cognitive decline with high reliability and correlates with a wide range of cognitive tests [32].

The *Lawton Brody IADL scale*—assesses performance in eight domains of IADLs (score range 0–8 women; 0–5 men), with higher scores indicating better performance [33]. To achieve comparability between subjects regardless of gender, in this study the scores were dichotomized into impaired = 1 (i.e. at least one considered activity with impairment) and not impaired = 0. The Lawton Brody IADL scale is one of the most frequently used IADL tools, with high reliability estimates. However, it has limitations due to content aspects (e.g. face validity), possibly due to its long existence [14, 19].

The *Depression in old Age Scale *(*DIA-S*)—is a relatively new screening tool to measure depression (score range 0–10); scores > 4 indicating probable pathological depression [34]. The DIA-S has been shown to have high discriminative power in terms of internal consistency and specificity compared to the Geriatric Depression Scale [35].

### Analysis

All analyses used the statistical software R (version 3.6.3) [36] and Mplus (version 7) [37]; the alpha level was set to 0.05.

Differences in the demographic characteristics of the included participants from the different settings (i.e. memory clinic setting, community) were investigated using Welch two sample *t*-test or Pearson’s Chi-square test, where appropriate.

The original A-IADL-Q-SV was fitted to a full graded response model on the basis of approximate marginal maximum likelihood estimation [22]. Unidimensionality of the A-IADL-Q-SV German was examined using Confirmatory Factor Analysis (CFA) through investigating the factor structure (one-factor model) [2, 22]. Model fit to the full graded response model of the A-IADL-Q-SV German was evaluated with the comparative fit index (CFI > 0.90) and root mean square error of approximation (RMSEA < 0.05), as described elsewhere [22]. To further examine unidimensionality, we calculated a difference approximation to the second-order derivatives along the scree plot based on eigenvalue decomposition on the matrix of robust Spearman correlations between the items [38]. The resulting acceleration approximation indicates points of abrupt change along the scree plot, and the number before the point with the maximum acceleration value indicates the number of latent dimensions [38]. We assessed local independence by inspecting the residual correlation matrices, and considered residual correlations > 0.25 as indicative of potentially problematic item pairs [22], and evaluated the monotonicity assumption using Mokken scale analysis [39]. Subsequently, measurement invariance was examined by means of differential item functioning (DIF) analysis for culture, comparing Swiss-German participants with the Dutch reference sample. The reference sample encompassed the participants from the Amsterdam Dementia Cohort (n = 699) [40]. No DIF, i.e. measurement invariance, in this context means that the items function identically in culturally different samples [41]. Uniform DIF is defined as a consistent difference between groups across the latent trait level, in this case IADL functioning. Non-uniform DIF occurs when an item is easier or more difficult for one group compared to the other at the same level of the latent trait [23]. Sufficient item endorsement, defined as at least two selected response categories per item, was required for DIF analysis [18]. The DIF analysis was based on ordinal logistic regressions: for every item a null model and three hierarchically nested models were created and compared. Statistically significant DIF was determined based on the likelihood-ratio chi-square test with an alpha level of 0.01. To detect practically meaningful DIF, a cut-off on the change in McFadden’s pseudo R^2^ of ≥ 0.035 was used [42]. We then performed Monte Carlo simulations over 100 replications to refine detection criteria as well as effect size measures. These are computed repeatedly over simulated data based on the empirical data sets [43]. For the DIF analyses, we used the ‘lordif’ package version 0.3-3, developed by Choi et al. [43].

Test–retest reliability was investigated on the scale level of the T-scores based on intraclass correlation coefficient (ICC) (ICC_3,1_, two-way mixed effects consistency model, single measurement) [44], overall and separately for the groups of participants with MCI/MD and with NC. The standard error of measurement (SEM) was calculated as the square root of the residual variance of the model and graphically depicted by a Bland and Altman plot [23]. Additionally, the smallest detectable change (SDM) was calculated using the formula ± 1.96 × √2 × SEM [23]. For interpretation of the SEM, it was compared to the total range of the T-scores (i.e. 20 to 80). Based on previous research on the A-IADL-Q [16, 17, 22] an SEM < 6 was interpreted as acceptable.

Construct validity was assessed by examination of Spearman’s correlations between the A-IADL-Q-SV German and age, education, the MMSE, CDR, IQCODE, Lawton Brody IADL Scale and DIA-S. Based on the results from previous studies on the A-IADL-Q, the hypotheses were stated quite specifically [15, 22, 45] (Table 2).

Discriminant validity was investigated to ascertain whether the A-IADL-Q-SV German version was able to discriminate between the three diagnostic groups of NC, MCI, MD. Differences in the T-scores between these groups were investigated using the Kruskall-Wallis rank sum test, followed by post hoc pairwise Wilcoxon tests. The Bonferroni-Holm method was applied to correct for multiple testing.

## Results

In total, 96 community-dwelling elderly people were included, 56 (58%) from memory clinics and 40 (42%) from the community. The mean age of participants was 73.5 years (range 60–86 years); 44 (46%) were female; and, for 93 (97%) of the participants the duration of their relationship with their informant was > 10 years. Participants recruited from memory clinics were older, had a lower level of education and were more impaired on the A-IADL-Q-SV German than participants recruited from the community. Informants of the participants from memory clinics were less often a spouse and more often children. They lived apart from their informants more often compared to the participants recruited from the community and their informants. Details of demographic and clinical characteristics are summarized in Table 1.

**Table 1:** Participant and informant characteristics

	Participants (n = 96)				Informants (n = 96)		
		Memory clinic (n = 56)	Community (n = 40)	*p* value	Memory clinic (n = 56)	Community (n = 40)	*p* value
Age	74.08 (6.77)	77.04 (6.13)	69.95 (5.34)	*p* < 0.001	64.73 (15.3)	64.5 (11.66)	*p* = 0.9
(range)	(60–89)	(62–89)	(60–83)		(30–89)	(34–83)	
Female (%)	44 (45.8%)	25 (44.6%)	19 (47.5%)	*p* = 0.9	38 (67.8%)	27 (67.5%)	*p* = 1
Level of education^a^	1; 7 (7%)	1; 6 (10%)	1; 1 (2.5%)	*p* < 0.01	1; 5 (11%)	1; 2 (5%)	*p* = 0.4
	2; 54 (57%)	2; 38 (67.5%)	2; 17 (42.5%)		2; 32 (57%)	2; 19 (47.5%)	
	3; 24 (25%)	3; 10 (18%)	3; 14 (35%)		3; 15 (27%)	3; 16 (40%)	
	4; 5 (5%)	4; 0 (0%)	4; 5 (12.5%)		4; 3 (5%)	4; 3 (7.5%)	
	5; 5 (5%)	5; 2 (3.5%)	5; 3 (7.5%)		5; 1 (2%)	5; 0	
Diagnosis		MCI 27 (48%)	NC 40 (100%)		NA	NA	
		MD 26 (46%)					
		NC 3 (6%)					
Relationship spouse					32 (57.1%)	31 (77.5%)	*p* < 0.01
Duration (> 10 years)					55 (98%)	38 (95%)	*p* = 0.7
Living together					32 (57%)	30 (75%)	*p* < 0.01
A-IADL-Q-SV							
T-score	59.89 (9.29)	54.71 (8.37)	67.13 (4.41)	*p* < 0.001			
Latent Trait score	− 0.99 (0.93)	− 0.47 (0.84)	− 1.71 (0.44)	*p* < 0.001			
Clinical measures							
MMSE		25.05 (2.94)	NA				
CDR Median (IQR)		0.5 (0.5)	NA				
IQCODE		3.69 (0.51)	NA				
Lawton Brody^b^		39 (74%)	NA				
DIA-S Median (IQR)		2 (3)	NA				
mTICS		NA	37.0 (3.75)				

Values are means (standard deviation), medians (interquartile range) or frequencies. *p*-values based on Welch two sample *t*-tests or Pearson’s Chi-square tests

*A-IADL-Q-SV* Amsterdam Instrumental Activities of Daily Living Questionnaire Short, *MMSE* Mini Mental State Examination, *CDR* Cumulative Dementia Rating, *IQCODE* Informant Questionnaire for Cognitive Decline in the Elderly, *DIA-S* Depression in old age scale, *mTICS* modified Telephone Interview for Cognitive Status, *MCI* mild cognitive impairment, *MD* mild dementia, *NC* normal cognition, *NA* not applicable

^a^Level of education in accordance to the international standard classification of education: ISCED (1 = ISCED 2, 2 = ISCED 3–5, 3 = ISCED 6, 4 = ISCED 7, 5 = ISCED 8)

^b^Dichotomized impaired/non impaired

### Measurement invariance

We checked the basic assumptions for IRT scoring. The Additional file 1: Table 1 provides the graded response model estimates for item parameters and item information values in the reference sample. The CFI showed a good model fit (0.95), but the RMSEA (0.11, 95% CI [0.10, 0.12]) was indicative for borderline poor model fit. Several items had high inter-item correlation, probably due to restricted response variation. All items loaded significantly on the IADL factor (one factor model), confirming unidimensionality. Furthermore, the maximum acceleration value from the scree plot was at the first factor, confirming unidimensionality. No items violated the monotonicity assumption. A few item pairs showed a potential local dependence, possibly due to restricted variability in item responses; details on these item pairs are presented in the Additional file 2: Table 2.

**Fig. 1:**
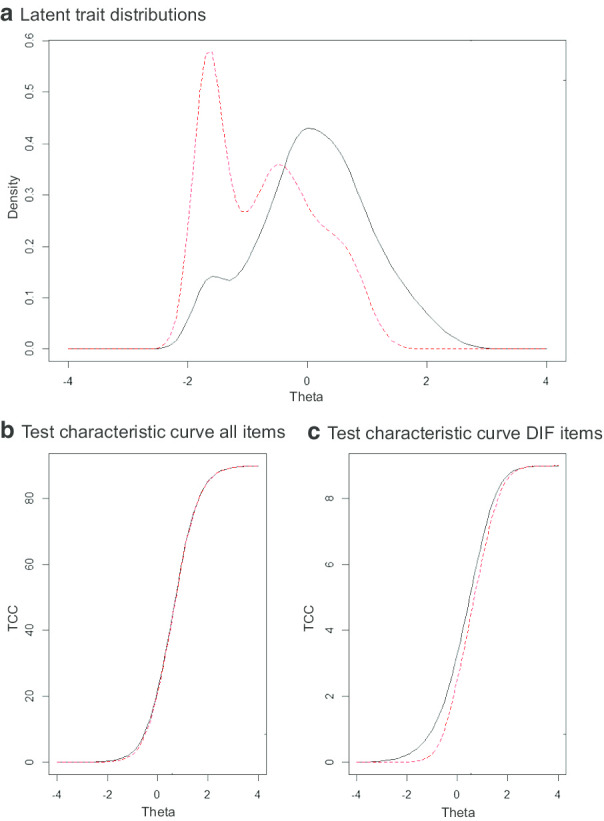
Latent trait distributions and test characteristic curves. The red dotted lines show the Swiss population and the solid black lines the Dutch reference group. **a** Latent trait distributions for the Dutch reference sample and the Swiss sample. **b** Test characteristic curves including all items for the Dutch reference sample and the Swiss sample. **c** Test characteristic curves including the three items with differential item functioning for the Dutch reference sample and the Swiss sample

Figure 1a shows the distributions of the trait (i.e. theta), Fig. 1b depicts the test characteristic curves for all items, and Fig. 1c the test characteristic curves for the items with DIF for the Swiss sample and the Dutch reference sample. All items were sufficiently endorsed by both groups. The results from the likelihood-ratio chi-square tests indicated three items with statistically significant DIF: item 2 ‘Doing the shopping’; item 20 ‘Working’; item 23 ‘Printing documents’; the item characteristic curves for these items are depicted in the Additional file 3: Figure 1. Items 2 and 23 showed uniform DIF, with item 2 being easier and item 23 being more difficult in the Swiss sample compared to the Dutch reference sample. Item 20 showed non-uniform DIF. However, effect sizes (change in McFadden’s pseudo R^2^) were negligible (i.e. R^2^ < 0.035; for item 2 R^2^ = 0.008, item 20 R^2^ = 0.02, item 23 R^2^ = 0.015), suggesting that there was no practically meaningful item bias. All chi-squared values and ΔR^2^ values for the logistic regressions obtained from the empirical data used for the DIF analyses are presented in the Additional file 4: Table 3. Monte Carlo simulations confirmed that the a priori cut-offs we used, were appropriate. The Monte Carlo simulations-based cut-off of chi-squared *p* values and ΔR^2^ values can be found in the Additional file 5: Table 4. We corrected for DIF by means of a re-estimation of the T-scores in the Swiss sample based on the DIF results. The mean T-score increased by 0.38 points, and the largest individual change was an increase of 2.17, corresponding to approximately one-fifth of a SD change.

### Test–retest reliability and measurement error

**Fig. 2:**
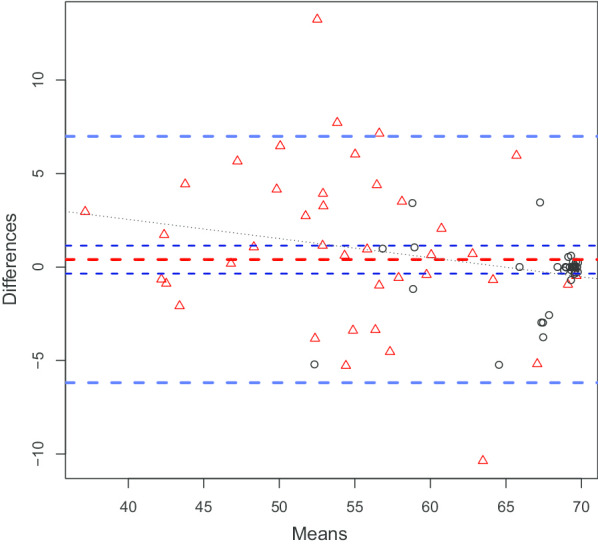
Bland and Altman plot including all participants. The X-axis shows the means of the T-scores of the two measurement time points and the Y-axis the differences in means of the T-scores between the two measurements. The horizontal red dashed line represent the mean difference, the dark blue dashed lines the 95% CI of the mean difference, the blue dashed lines the lower and upper limits of agreement, and the black dotted line the regression line between the mean of the T-scores and difference in the means of the T-scores. Triangles represent participants with mild cognitive impairment or mild dementia and circles participants with normal cognition

Of the included 96 informants, 82 (85%) completed the A-IADL-Q-SV German for the second time, with a median of 23 days between the two measurement time points; two questionnaires were excluded because a different informant had completed the second questionnaire, resulting in the inclusion of 80 questionnaires in the analysis. An overall ICC of 0.93 (95% CI [0.9, 0.96]) was observed. SEM, by means of classical test theory, was 2.4 and the smallest detectable change was 6.6 (95%CI [5.3, 7.9]). The range of the T-Score was 39.7. The corresponding Bland and Altman Plot is depicted in Fig. 2. The mean difference between the two measurements was 0.4 (95% CI [− 0.4, 1.2], *p* = 0.29); the lower limit of agreement was − 6.2 (95% CI [− 7.5, − 4.9]) and the upper limit of agreement 7 (95% CI [5.7, 8.3]). The Bland and Altman plot shows that the data for the group of participants with NC (higher level of IADL functioning) has less variance. Furthermore, residual analysis showed that the data did not conform to model assumptions (i.e. homoscedasticity and normal distribution of residuals).

**Fig. 3:**
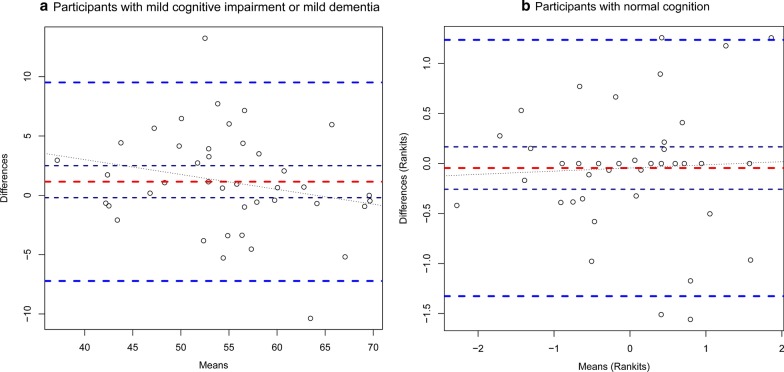
Bland and Altman plot of the subgroups. The horizontal red dashed line represent the mean difference, the dark blue dashed lines the 95% CI of the mean difference, the blue dashed lines the lower and upper limits of agreement, and the black dotted line the regression line between the mean and difference in the means. **a** The X-axis shows the means of the T-scores of the two measurement time points and the Y-axis the differences in means of the T-scores between the two measurements. **b** Note this figure is based on the rankits of the T-score in participants with normal cognition. The X-axis shows the means of rankits of the T-scores of the two measurement time points and the Y-axis the differences in means of rankits of the T-scores between the two measurements

The separate ICCs for the subgroups of participants with MCI/MD (n = 41) and with NC (n = 39) were also estimated. For the MCI/MD subgroup, an ICC of 0.86 (95% CI [0.77, 0.91]) was observed, compared to the NC subgroup with an ICC of 0.92 (95% CI [0.86, 0.95]). Subsequently, the SEM, SDC and Bland and Altman analyses for the subgroups of participants with MCI/MD and NC participants were investigated separately. The SEM in the MCI/MD subgroup was 3 and the SDC 8.4 (95% CI [6.1, 8.4]). The Bland and Altman Plot for the MCI/MD subgroup is depicted in Fig. 3a. There was no evidence of violation of model assumptions. The mean difference of the two measurements was 1.1 (95% CI [− 0.2, 2.5], *p* = 0.93); the lower limit of agreement was − 7.2 (95%CI [− 9.6, − 4.9]) and the upper limit of agreement 7 (95% CI [7.2, 11.9]). As in the NC subgroup, approximative normality of differences based on residual analyses could not be confirmed, so the T-scores were transformed into rankits (i.e. standard normal deviates of the corresponding rank) [23]. The SEM based on the rankit-transformed T-scores was 0.46 and the SDC 1.3 (95% CI [− 1.3, 1.2]). The corresponding Bland and Altman Plot is depicted in Fig. 3b. The mean difference of the rankits of the two measurements was − 0.05 (95% CI [− 0.3, 0.2], *p* = 0.17); the lower limit of agreement was − 1.3 (95% CI [− 1.7, − 0.96]) and the upper limit of agreement 1.2 (95% CI [0.9, 1.6]).

### Construct validation of the A-IADL-Q-SV-G

Point estimates of the observed correlations between the A-IADL-Q-SV German and education based on the CDR, IQCODE, Lawton Brody Scale and MMSE were in the direction and of the magnitude hypothesized. Age was more strongly associated with the A-IADL-Q-SV German than hypothesized [− 0.39, 95% CI (− 0.60 to − 0.15)] and point estimates for depression were in the opposite direction. All hypothesized and observed correlations are summarized in Table 2.

**Table 2:** Construct validation Spearman’s correlation coefficients of T-Scores of the A-IADL-Q-SV German with clinical measures

Measure	Hypothesized correlations		n	Observed correlations [95% CI]	
	Direction	Range			
Age	−	0.0–0.2	56	− 0.39	[− 0.60, − 0.15]
Education^a^	+	0.0–0.2	56	0.07	[− 0.19, − 0.33]
Everyday functioning					
CDR	−	0.2–0.4	56	− 0.35	[− 0.56, − 0.09]
IQCODE	−	0.4–0.7	53	− 0.69	[− 0.81, − 0.51]
Lawton Brody scale^b^	−	0.4–0.7	53	− 0.41	[− 0.61, − 0.16]
Cognitive function					
MMSE	+	0.2–0.4	56	0.38	[− 0.13, − 0.58]
Depression					
DIA-S	−	0.0–0.2	53	0.01	[− 0.028, − 0.27]

*CDR* Cumulative Dementia Rating, *IQCODE* Informant Questionnaire for Cognitive Decline in the Elderly, *MMSE* Mini Mental State Examination, *DIA-S* Depression in old age scale

^a^Level of education in accordance with the international standard classification of education: ISCED (1 = ISCED 2, 2 = ISCED 3–5, 3 = ISCED 6, 4 = ISCED 7, 5 = ISCED 8)

^b^Dichotomized impaired = 1/non impaired = 0

### Discriminant validity

**Fig. 4:**
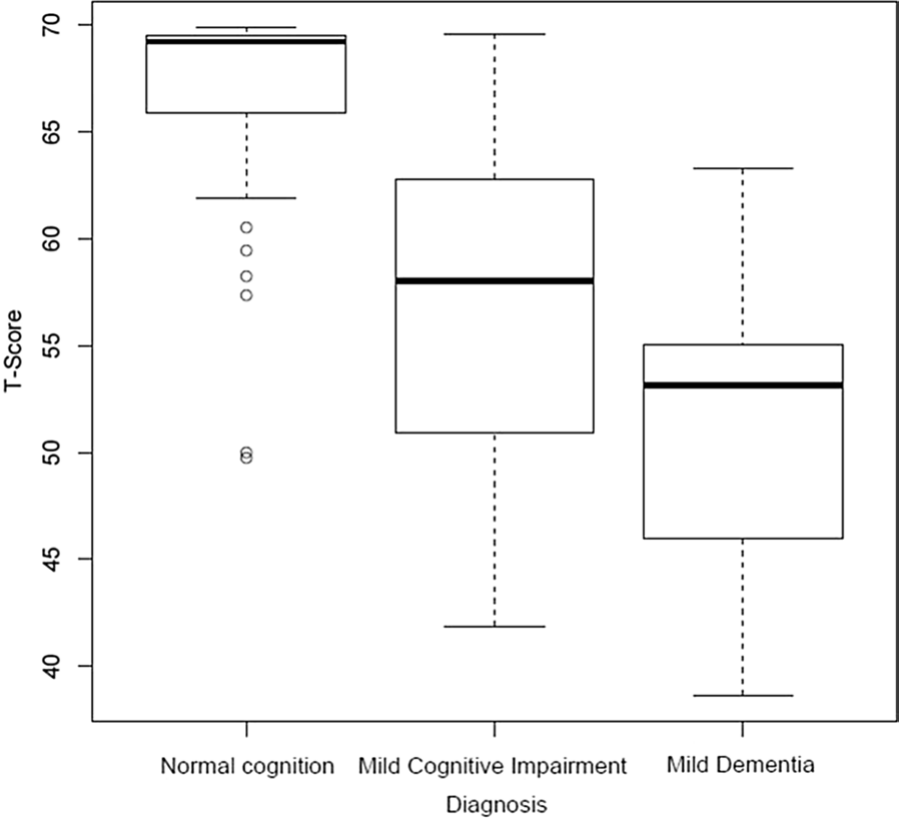
T-Scores of the three diagnostic groups

Figure 4 shows the mean of the T-scores for participants with NC as 67 (range 50–70), those with MCI as 57 (range 42–70) and those with MD as 51 (range 39–63). Homogeneity of variances could not be assumed based on Levene’s test for homogeneity of variances (F-value 6.54, *df* = 2, *p* value = 0.0022) and Bartlett test of homogeneity of variances (Bartlett’s K-squared = 10, *df* = 2, *p* value = 0.008). Therefore, non-parametric analyses were performed. The results derived from the Kruskal–Wallis rank sum test indicated that the location parameters of the T-scores between the three diagnostic groups differed (Kruskal–Wallis Chi-square = 49, *df* = 2, *p* < 0.001). Post-hoc pairwise comparisons using Wilcoxon rank sum tests revealed the following differences: NC versus MCI (*p* < 0.001); NC versus MD (*p* < 0.001); and MCI versus MD (*p* < 0.05).

## Discussion

The results of the cross-cultural adaptation and validation indicated that the A-IADL-Q-SV German retained the measurement properties, i.e. there was no evidence of measurement invariance by means of DIF, good construct validity, discriminant validity and test–retest reliability of the original version in a Swiss-German population of elderly people with NC, MCI and MD. Therefore, the A-IADL-SV German has been shown to be a psychometrically robust measurement instrument to assess IADL in elderly people within the range of no cognitive impairment to mild dementia. It is also comparable across countries.

In terms of measurement invariance by means of DIF, all basic assumptions for IRT scoring were met. This is in line with previous research on the A-IADL-Q-SV, indicating that the questionnaire measures one construct (i.e. IADL functioning) [22]. The high inter-item correlations, which may have influenced the model fit indices, might be a reflection of the inclusion of less impaired participants in the Swiss-German sample compared to the Dutch reference sample. Our sample included participants from NC to MD, compared to the Dutch reference sample that included only memory clinic patients, who were generally more IADL impaired. In the Swiss-German sample, a high proportion of people rated most of the items with ‘no problems’, which may have inflated the inter-item correlations. A few item pairs (1%) showed larger correlation residuals than > 0.25. This may indicate that the local independence of these items is compromised. The large residuals may also be caused by the fact that the sample was relatively homogeneous with regards to their level of overall functional impairment. This caused a limited variability in selected item responses. As most residuals are only marginally above the cut-off, and because other analyses show that the original IRT model fits and provides reliable estimates of everyday functioning, we are confident, that the IRT model appears to fit in the Swiss sample.

The results of DIF analysis based on empirical data using pre-defined cut-offs indicated that the A-IADL-Q-SV German was robust to differences between the Swiss and Dutch cultures. Due to the small sample size in the Swiss sample we additionally used Monte Carlo simulation to obtain the 99th percentile of the most extreme chi-squared values and ΔR2 values under the assumption that there is no DIF. The Monte Carlo simulations thus provide more precise cut-offs for the chi-squared test and ΔR2 values. The items flagged for DIF using a priori thresholds matched the items flagged using the Monte Carlo thresholds, providing support for the a priori thresholds. The findings of the DIF analysis agree with a previous investigation on item bias in eight Western countries, which indicated that the A-IADL-Q-SV was robust to cultural differences, as well as to age, sex and education [18].

In terms of test–retest reliability on scale level, our results indicated a good to excellent ICC based on a two-way mixed effects consistency model overall, as well as in the MCI/MD subgroup and NC subgroup. Previous research investigated test–retest reliability of the A-IADL-Q on the item level and revealed high test–retest reliability [2]. However, test–retest reliability of the A-IADL-Q-SV on scale level has not been investigated previously. Nonetheless, the results of test–retest reliability on scale level of the A-IADL-Q-SV is relevant for clinical and research purposes. Both aim to use the questionnaire as an outcome measure, since the total score is interpreted [41].

The SEM overall, as well as in the MCI/MD and NC subgroups, calculated by means of classical test theory, are implied to be acceptable with reference to the range of the T-Scores. Measurement error was also investigated with Bland and Altman analyses. We observed that the data for the subgroup of participants with NC did not conform to the assumptions of the model. Therefore, we transformed the T-Scores into rankits to rerun the analysis. The results of the Bland and Altman analyses overall and in the subgroups indicated that a change in the T-scores of more than eight points might be interpreted as real change [23].

Construct validity in terms of hypothesis testing was shown, with more than 75% of the stated hypotheses being confirmed [41]. The hypotheses were specifically stated based on previous research on the A-IADL-Q [15] and A-IADL-Q-SV [22, 45]. The correlations between the A-IADL-Q-SV and the clinical measures of cognition and functioning were in the magnitude and direction as hypothesized and are, therefore, in line with previous studies [15, 22]. However, we observed a moderate correlation between the A-IADL-Q-SV German and age, whilst the original A-IADL-Q and A-IADL-Q-SV observed small correlations [15, 18, 22]. Another study on the A-IADL-Q in Spain also observed a moderate correlation of the A-IADL-Q with age [45]. The findings in our study might be explained by the significantly higher age of participants with MCI and MD (and hence with significantly more IADL limitations) than participants with NC. A study investigating age as a source of item bias on the A-IADL-Q-SV found that the T-scores were not influenced by age [18].

Furthermore, a positive correlation between the A-IADL-Q-SV German and the DIA-S was observed, which stands in contrast to our hypothesis and previous research [15, 22]. This may be due to the different measurement instruments used to assess depression. The DIA-S was developed to assess depression in accordance with the diagnostic criteria of depression and, therefore, includes different items than those on the geriatric depression scale (GDS). Only a moderate correlation was observed between the DIA-S and GDS [34]. However, since the observed correlation in our study between depression and IADL limitation was small, and in line with the literature [15, 22], it may be concluded that IADL limitation, as measured by the A-IADL-Q-SV, is not influenced by depression.

In terms of discriminant validity our results indicate that the A-IADL-Q-SV German was able to discriminate between participants with NC, MCI and MD. The interpretation of T-scores observed in our study fitted well with the interpretation scheme. In fact, a previous study investigating the diagnostic value of the A-IADL-Q found a cut-off of 51.4 to differentiate between people with dementia and people without dementia [16], corresponding almost perfectly to the mean T-score found in our MD group.

### Limitations

We acknowledge some limitations to our study. A major limitation of this study may be the sample size. In terms of test–retest reliability based on ICC’s and estimates of measurement error, the number of participants was relatively small in the two subgroups. This is reflected by the 95% CI of the ICCs of the subgroups (width > 0.2) and the change of the limits of agreement between the overall sample and the subgroup of participants with MCI/MD. With respect to the investigation of construct validity based on hypothesis testing, the small sample (n = 56) may have produced wide confidence intervals. A larger sample would have provided more precise estimates of the correlations. Furthermore, the overall sample size may have been too small to detect subtle measurement invariance with DIF analysis. However, the ordinal logistic regression approach used in our study has previously been shown to be capable of detecting DIF when the reference sample is large, even when the focus sample is smaller [46]. Nonetheless, the generalizability of our results may be limited due to the restricted sample size.

Participants with NC were recruited from the community, while participants with MCI and MD were recruited from memory clinics associated with geriatric institutions, using a convenient sampling strategy. This may have produced bias that is reflected in the differences in demographics.

Cognitive status for participants with NC was investigated solely using TICS-m, a telephone screening for cognitive decline. Therefore, the possibility of participants with, so-called, subjective cognitive decline also being included in this group cannot be ruled out.

Information on participants’ comorbidities was collected restrictively, meaning that the chance of comorbidities having influenced our results also cannot be excluded. However, due to its scoring structure, the A-IADL-Q-SV considers only those limited activities related to cognitive problems. Furthermore, participants with comorbidities known to have an influence on cognitive function were excluded (i.e. clinical depression, drug and alcohol abuse, as well as neurological diseases, such as Parkinson’s disease, stroke or traumatic brain injuries). Finally, data on the factors known to influence IADL functioning were collected, i.e. age, sex, level of education and living situation. As a result, we are convinced that the A-IADL-Q-SV T-Scores correctly represent the level of IADL functioning, controlled for, e.g. physical impairments.

A subgroup of cognitively healthy participants was included in the test–retest analysis and in the investigation of measurement error. This inclusion of less-impaired participants may have inflated the overall ICC, because the heterogeneity of the overall sample was increased. Consequently, test–retest reliability and measurement error in the subgroups were also investigated separately. However, the inclusion of such participants was relevant for our study, because the decline in IADL functioning from a previously measured level often predates cognitive decline [8].

Finally, our sample was not severely impaired and does not reflect the full dementia spectrum. Future investigations of the A-IADL-Q-SV German should use larger samples and include younger patients with MCI or a dementia diagnosis, as well as participants at the later stages of cognitive decline, i.e. moderate dementia and severe dementia.

## Conclusion

The cross-culturally validated A-IADL-Q-SV German has retained the psychometric properties (i.e. measurement invariance, test–retest reliability, construct validity and discriminant validity) of the original version. This study implies that the A-IADL-Q-SV German is a promising tool for use in clinical practice to investigate IADL functioning in elderly people with normal cognition, mild cognitive impairment and mild dementia. It is also useful for research purposes and allows international comparisons to be made.

## Supplementary information

**Additional file 1: Table 1.** GRM Item parameters and item information values. Item parameter and item information values estimated in the reference sample used for differential item functioning detection in the Swiss sample. Item parameters are shown as parameter ± standard error. Abbreviations: GRM, Graded Response Model; α, discrimination parameter; β’s, extremity parameters.

**Additional file 2: Table 2.**Investigation of local independence. Item pairs with large residuals (> 0.25) in the one-factor model fit

**Additional file 3: Figure 1.** Item characteristic curves.

**Additional file 4: Table 3.** Differential Item Functioning from empirical data. Chi-square and McFadden’s ΔR^2^ values as obtained in differential item functioning (DIF) analyses from the empirical data. Items flagged for DIF are displayed italic in blue. Empirical cut-offs were set a priori at *α* < .01 for statistically significant DIF, and ΔR^2^ > .035 for clinically meaningful DIF.

**Additional file 5: Table 4.** Differential Item Functioning Monte Carlo Simulations. The values displayed represent the 99^th^-percentile threshold values for chi-square *p*-values and McFadden’s ΔR^2^ values, obtained from Monte Carlo simulations under the assumption that there is no DIF. When the values found in the empirical data set are more extreme (i.e., smaller *p*-value and larger ΔR^2^ value) than those found in the Monte Carlo simulations, this suggests there is DIF. Items flagged for DIF are displayed italic in blue.

## Data Availability

The datasets used and/or analysed during the current study are available from the corresponding author on reasonable request.

## Abbreviations

A-IADL-Q: Amsterdam Instrumental Activities of Daily Living questionnaire
A-IADL-Q-SV: Amsterdam Instrumental Activities of Daily Living questionnaire short version
CDR: Clinical Dementia Rating
CFI: Comparative fit index
CFA: Confirmatory factor analysis
DIA-S: Depression in old age scale
DIF: Differential item functioning
GDS: Geriatric depression scale
IADL: Instrumental Activities of Daily Living
IQCODE: The Informant Questionnaire for Cognitive Decline in the Elderly
IRT: Item response theory
ISCED: International standard classification of education
MCI: Mild cognitive impairment
MD: Mild dementia
MMSE: Mini Mental State examination
TICS-m: Modified telephone interview for cognitive status
NC: Normal cognition
SDC: Smallest detectable change
SEM: Standard error of measurement
