# Supplementary material for: Cross-cultural adaptation and validation of the Amsterdam Instrumental Activities of Daily Living questionnaire short version German for Switzerland
Source: Health Qual Life Outcomes. 2020 Oct 2;18:323. doi: 10.1186/s12955-020-01576-w (PMC7530958; doi:10.1186/s12955-020-01576-w)
Supplement: Supplementary file 1 — Additional file 1: Table 1. GRM Item parameters and item information values. Item parameter and item information values estimated in the reference sample used for differential item functioning detection in the Swiss sample. Item parameters are shown as parameter ± standard error. Abbreviations: GRM, Graded Response Model; α, discrimination parameter; β’s, extremity parameters. [file 12955_2020_1576_MOESM1_ESM.pdf]

Additional table 1 – GRM Item parameters and item information values

Item no.	Item	Item parameters α	β1	β2	β3	β4	Item information
Q1	Carrying out household duties	1.75±0.09	-0.31±0.06	0.83±0.14	1.82±1.04	2.81±8.15	4.61
Q2	Doing the shopping	2.35±0.12	-0.33±0.05	0.67±0.11	1.42±0.95	1.96±4.90	6.10
Q3	Buying the correct articles	2.06±0.11	-0.15±0.05	0.85±0.17	1.32±0.78	1.45±2.23	4.11
Q4	Cooking	2.30±0.12	-0.20±0.05	0.75±0.14	1.28±0.79	1.73±2.82	5.40
Q5	Preparing sandwich meals	1.94±0.13	1.05±0.06	1.84±1.64	2.49±8.40	2.89±30.47	4.14
Q6	Making minor repairs to the house	2.58±0.13	-0.48±0.05	0.32±0.05	0.76±0.26	1.01±0.73	5.62
Q7	Operating domestic appliances	2.29±0.13	0.32±0.05	1.27±0.55	1.99±3.98	2.58±22.59	5.85
Q6	Operating the microwave	2.01±0.12	0.69±0.05	1.36±0.67	1.88±2.57	2.08±7.32	3.79
Q9	Operating the coffee maker	1.90±0.13	1.05±0.06	2.01±1.99	2.49±9.67	2.71±26.54	3.78
Q10	Operating the washing machine	1.91±0.12	0.91±0.06	1.73±1.20	2.12±4.65	2.18±15.26	3.36
Q11	Paying bills	3.50±0.19	-0.01±0.04	0.60±0.18	0.90±0.83	1.13±2.26	7.76
Q12	Using a mobile phone	2.02±0.11	-0.04±0.05	0.90±0.19	1.64±1.17	2.37±6.58	5.03
Q13	Managing the paperwork	3.32±0.18	-0.54±0.04	0.34±0.04	0.72±0.34	0.96±1.05	8.06
Q14	Using electronic banking	2.96±0.16	-0.01±0.04	0.66±0.17	0.94±0.72	1.25±1.88	6.35
Q15	Using a PIN code	1.93±0.12	0.63±0.05	1.44±0.65	1.87±2.55	2.14±6.59	3.73
Q16	Obtaining money from an ATM	2.13±0.14	0.90±0.05	1.51±1.11	1.75±3.50	1.88±7.37	3.56
Q17	Paying using cash	1.98±0.13	0.96±0.06	1.74±1.36	2.17±5.51	2.56±15.43	3.97
Q18	Making appointments	2.01±0.11	-0.33±0.05	0.50±0.08	1.20±0.46	1.64±1.63	4.49
Q19	Filling in forms	2.63±0.13	-0.62±0.05	0.43±0.05	0.91±0.39	1.27±1.26	6.42
Q20	Working	1.52±0.09	-1.06±0.08	-0.05±0.06	0.47±0.12	0.74±0.29	2.83
Q21	Using a computer	2.68±0.14	-0.33±0.05	0.56±0.09	1.07±0.62	1.60±2.77	6.84
Q22	Emailing	2.68±0.15	0.07±0.04	0.79±0.22	1.11±0.91	1.30±2.23	5.43
Q23	Printing documents	2.48±0.15	0.26±0.04	0.86±0.28	1.15±0.95	1.31±2.12	4.53
Q24	Operating devices	2.71±0.15	-0.01±0.04	1.10±0.45	1.85±4.96	2.29±27.15	7.43
Q25	Operating the remote control	1.61±0.10	0.37±0.06	1.53±0.48	2.53±3.46	3.20±18.10	3.85
Q26	Playing card and board games	1.23±0.08	-0.29±0.07	1.12±0.18	1.89±0.81	2.30±2.21	2.46
Q27	Driving a car	1.53±0.09	-0.22±0.06	0.83±0.14	1.24±0.53	1.52±1.14	2.83
Q28	Using a sat-nav system	2.13±0.12	-0.13±0.05	0.75±0.15	1.09±0.62	1.40±1.39	4.28
Q29	Using public transportation	2.11±0.12	0.21±0.05	1.05±0.30	1.58±1.39	1.84±4.06	4.37
Q30	Being responsible for own medication	1.50±0.09	0.15±0.06	1.17±0.26	2.09±1.36	2.84±6.17	3.39

Item parameters and item information values estimated in the Dutch reference sample used for differential item functioning detection in the Swiss sample. Item parameters are shown as parameter ± standard error

Abbreviations: GRM, Graded Response Model; α, discrimination parameter; β's, extremity parameters
