# Supplementary material for: Cross-cultural adaptation and validation of the Amsterdam Instrumental Activities of Daily Living questionnaire short version German for Switzerland
Source: Health Qual Life Outcomes. 2020 Oct 2;18:323. doi: 10.1186/s12955-020-01576-w (PMC7530958; doi:10.1186/s12955-020-01576-w)
Supplement: Supplementary file 2 — Additional file 2: Table 2.Investigation of local independence. Item pairs with large residuals (> 0.25) in the one-factor model fit [file 12955_2020_1576_MOESM2_ESM.pdf]

Additional table 2 – Investigation of local independence

Item 1	Item 2	Residual
Q 8	Q13	-0.279
Q 9	Q16	-0.258
Q10	Q16	-0.367
Q14	Q17	0.252
Q15	Q23	-0.273

Item pairs with large residuals (> 0.25) in the one-factor model fit
