# Supplementary material for: Cross-cultural adaptation and validation of the Amsterdam Instrumental Activities of Daily Living questionnaire short version German for Switzerland
Source: Health Qual Life Outcomes. 2020 Oct 2;18:323. doi: 10.1186/s12955-020-01576-w (PMC7530958; doi:10.1186/s12955-020-01576-w)

Additional figure 1 – Item characteristic curves

a) Item characteristic curve Item 2

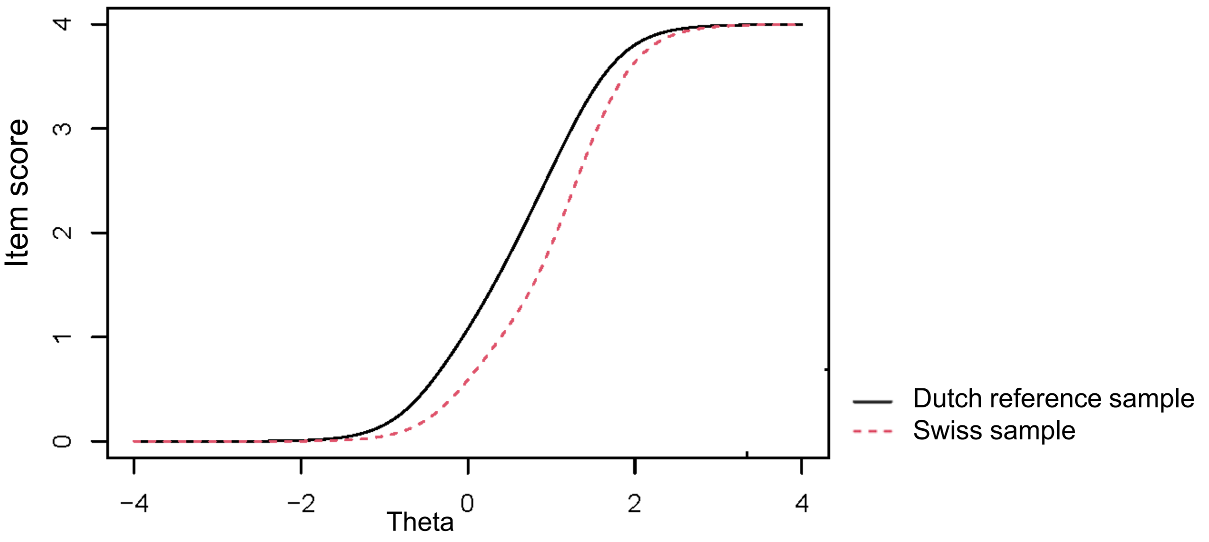

b) Item characteristic curve Item 20

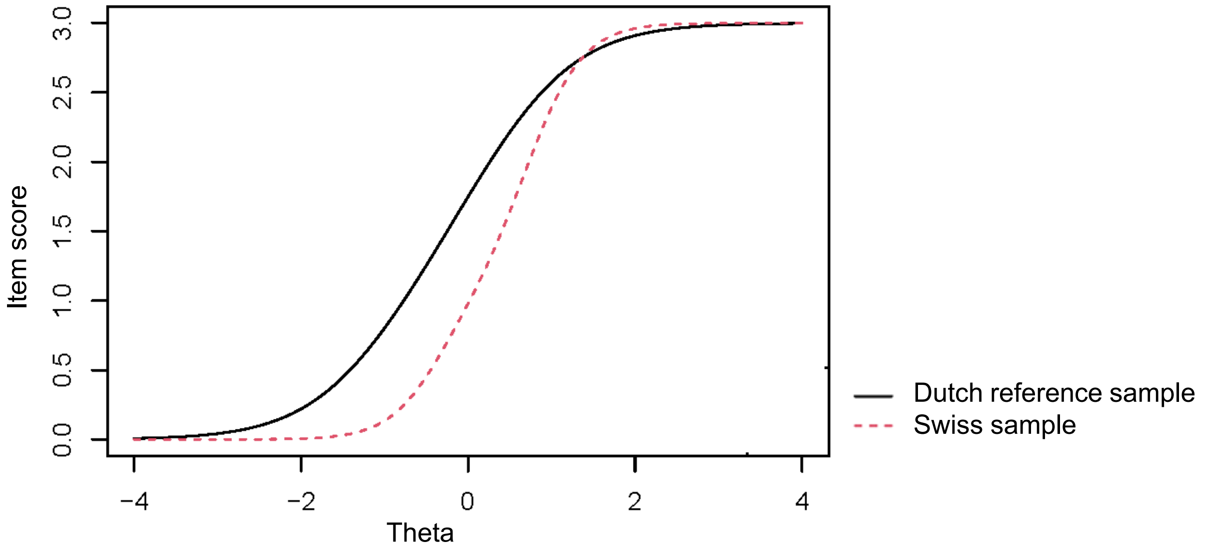

c) Item characteristic curve Item 23

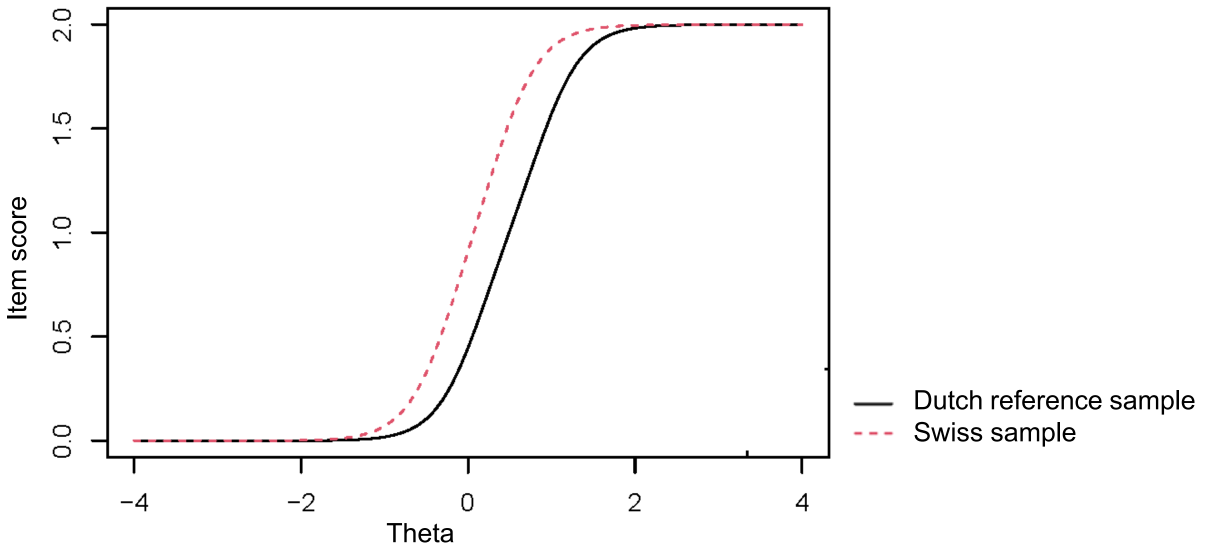

Supplement: Supplementary file 3 — Additional file 3: Figure 1. Item characteristic curves. [file 12955_2020_1576_MOESM3_ESM.pdf]
