# Supplementary material for: Cross-cultural adaptation and validation of the Amsterdam Instrumental Activities of Daily Living questionnaire short version German for Switzerland
Source: Health Qual Life Outcomes. 2020 Oct 2;18:323. doi: 10.1186/s12955-020-01576-w (PMC7530958; doi:10.1186/s12955-020-01576-w)
Supplement: Supplementary file 4 — Additional file 4: Table 3. Differential Item Functioning from empirical data. Chi-square and McFadden’s ΔR2 values as obtained in differential item functioning (DIF) analyses from the empirical data. Items flagged for DIF are displayed italic in blue. Empirical cut-offs were set a priori at α < .01 for statistically significant DIF, and ΔR2 > .035 for clinically meaningful DIF. [file 12955_2020_1576_MOESM4_ESM.pdf]

Additional table 3 – Differential Item Functioning based on empirical data

Item	Chi-Square *p*-Value	Mcfadden's ΔR^2
Q1	0.239	0.001
**Q2**	**0.001**	**0.008**
Q3	0.356	0.002
Q4	0.357	< 0.001
Q5	0.844	< 0.001
Q6	0.632	< 0.001
Q7	0.288	0.001
Q8	0.921	< 0.001
Q9	0.597	< 0.001
Q10	0.535	0.002
Q11	0.942	< 0.001
Q12	0.926	< 0.001
Q13	0.272	0.002
Q14	0.072	0.005
Q15	0.404	< 0.001
Q16	0.097	0.007
Q17	0.802	< 0.001
Q18	0.422	< 0.001
Q19	0.133	< 0.001
**Q20**	**< 0.001**	**0.019**
Q21	0.265	0.001
Q22	0.067	0.003
**Q23**	**0.002**	**0.015**
Q24	0.105	0.003
Q25	0.183	0.002
Q26	0.137	0.002
Q27	0.905	< 0.001
Q28	0.364	< 0.001
Q29	0.888	< 0.001
Q30	0.320	< 0.001

Chi-square and McFadden's ΔR^2 values as obtained in differential item functioning (DIF) analyses from the empirical data. Items flagged for DIF are displayed italic in blue. Empirical cut-offs were set a priori at $\alpha < .01$ for statistically significant DIF, and $\Delta R^2 > .035$ for clinically meaningful DIF
