# Supplementary material for: Cross-cultural adaptation and validation of the Amsterdam Instrumental Activities of Daily Living questionnaire short version German for Switzerland
Source: Health Qual Life Outcomes. 2020 Oct 2;18:323. doi: 10.1186/s12955-020-01576-w (PMC7530958; doi:10.1186/s12955-020-01576-w)
Supplement: Supplementary file 5 — Additional file 5: Table 4. Differential Item Functioning Monte Carlo Simulations. The values displayed represent the 99th-percentile threshold values for chi-square p-values and McFadden’s ΔR2 values, obtained from Monte Carlo simulations under the assumption that there is no DIF. When the values found in the empirical data set are more extreme (i.e., smaller p-value and larger ΔR2 value) than those found in the Monte Carlo simulations, this suggests there is DIF. Items flagged for DIF are displayed italic in blue. [file 12955_2020_1576_MOESM5_ESM.pdf]

Additional table 4 - Differential Item Functioning Monte Carlo Simulations

**Item**	**Chi-Square *p*-Value**	**Mcfadden's ΔR^2**
Q1	0.001	0.006
**Q2**	**0.001**	**0.007**
Q3	0.001	0.007
Q4	0.004	0.007
Q5	0.016	0.009
Q6	0.009	0.004
Q7	0.006	0.007
Q8	0.001	0.009
Q9	0.034	0.006
Q10	0.001	0.010
Q11	0.001	0.006
Q12	0.002	0.006
Q13	0.010	0.004
Q14	0.023	0.005
Q15	0.016	0.005
Q16	0.001	0.016
Q17	0.001	0.010
Q18	0.015	0.004
Q19	0.033	0.003
**Q20**	**0.004**	**0.005**
Q21	0.016	0.004
Q22	0.002	0.006
**Q23**	**0.007**	**0.006**
Q24	0.017	0.004
Q25	0.006	0.006
Q26	0.028	0.004
Q27	0.008	0.004
Q28	0.003	0.006
Q29	0.014	0.005
Q30	0.002	0.006

The values displayed represent the 99th-percentile threshold values for chi-square *p*-values and McFadden's ΔR^2 values, obtained from Monte Carlo simulations under the assumption that there is no DIF. When the values found in the empirical data set are more extreme (i.e., smaller *p*-value and larger ΔR^2 value) than those found in the Monte Carlo simulations, this suggests there is DIF. Items flagged for DIF are displayed italic in blue.
